# AAPM medical physics practice guideline 6.a.: Performance characteristics of radiation dose index monitoring systems

**DOI:** 10.1002/acm2.12089

**Published:** 2017-05-12

**Authors:** Dustin A. Gress, Renee L. Dickinson, William D. Erwin, David W. Jordan, Robert J. Kobistek, Donna M. Stevens, Mark P. Supanich, Jia Wang, Lynne A. Fairobent

**Affiliations:** ^1^ Department of Imaging Physics University of Texas MD Anderson Cancer Center Houston TX USA; ^2^ Colorado Associates in Medical Physics Colorado Springs CO USA; ^3^ Department of Radiology University Hospitals Cleveland Medical Center Case Western Reserve University Cleveland OH USA; ^4^ National Physics Consultants, Ltd. Mentor OH USA; ^5^ Northwest Permanente, PC Kaiser Sunnyside Medical Center Clackamas OR USA; ^6^ Department of Diagnostic Radiology and Nuclear Medicine Rush University Medical Center Chicago IL USA; ^7^ Environmental Health and Safety Stanford University Stanford CA USA; ^8^ American Association of Physicists in Medicine Alexandria VA USA

## Abstract

The American Association of Physicists in Medicine (AAPM) is a nonprofit professional society whose primary purposes are to advance the science, education and professional practice of medical physics. The AAPM has more than 8,000 members and is the principal organization of medical physicists in the United States.

The AAPM will periodically define new practice guidelines for medical physics practice to help advance the science of medical physics and to improve the quality of service to patients throughout the United States. Existing medical physics practice guidelines will be reviewed for the purpose of revision or renewal, as appropriate, on their fifth anniversary or sooner.

Each medical physics practice guideline represents a policy statement by the AAPM, has undergone a thorough consensus process in which it has been subjected to extensive review, and requires the approval of the Professional Council. The medical physics practice guidelines recognize that the safe and effective use of diagnostic and therapeutic radiology requires specific training, skills, and techniques, as described in each document. Reproduction or modification of the published practice guidelines and technical standards by those entities not providing these services is not authorized.

The following terms are used in the AAPM practice guidelines:

•Must and Must Not: Used to indicate that adherence to the recommendation is considered necessary to conform to this practice guideline.
•Should and Should Not: Used to indicate a prudent practice to which exceptions may occasionally be made in appropriate circumstances.

## 1 Introduction

Radiation dose index monitoring (RDIM) systems may generally be identified as software that retrospectively collects radiation dose indices (RDI) and other acquisition parameters from imaging studies that use ionizing radiation, and stores those indices in a relational database along with patient demographics. The software typically includes a graphical user interface, which allows the end user to visualize RDI by study type, patient or other category for quality assurance or patient‐ or study‐specific investigations. When utilizing data from these RDIM systems, it is important to understand the applications and limitations of the recorded dose indices.^1^ At this time, none of the RDI represent location‐specific absorbed dose in an individual patient. Most are related to X‐ray beam output or X‐ray absorption at the image receptor. Software indications of organ absorbed doses and effective dose are based on standardized models of the human which incorporate organ‐ and tissue‐weighting factors adopted by ICRP^2^ and do not accurately represent the absorption or risk characteristics of any single individual. Standardized human models do not account for the variation observed in size and location of organs within normal individuals, do not account for disease processes, and might not match the age or gender of the patient. Consequently, there are significant limitations to the utility of absorbed and effective dose estimates, detailed discussion of which is beyond the scope of this document. The most significant limitations can be coarsely and briefly summarized as follows: Effective dose does not apply to individuals, and the current state of organ dose calculations in commercial RDIM software is not patient‐specific.

It is therefore the recommendation of this Medical Physics Practice Guideline (MPPG) that estimated organ and effective dose values must only be used with the direction and involvement of a Qualified Medical Physicist, and with careful consideration and understanding of limitations of the quantities. Furthermore, these estimated organ and effective dose values should not be included in physician‐dictated reports.

### 1.A Overview

Many facilities are implementing RDIM software systems in part due to increasing public concern over the use of ionizing radiation in diagnostic examinations, in light of radiation injuries from diagnostic imaging equipment,^3^, ^4^, ^5^ and to comply with applicable state regulations and accreditation requirements. The RDIM systems currently available from several vendors vary widely in their capabilities, degree of difficulty in implementing, ease of customizing the analyses of the data, and ability to grow with the change in clinical practice, imaging equipment, or regulatory requirements. It is therefore recommended that a facility considering the acquisition of a RDIM system create desired performance criteria just as for any other major piece of software such as voice recognition systems or picture archiving and communications systems (PACS). This practice guideline is intended to provide a guide to the minimum general and modality‐specific requirements for RDIM systems.

Before embarking on an effort to develop or install an RDIM system, it is essential to identify how the data will be used, who will oversee and support the facility's RDIM effort, who will have access to the software and its data, and at what levels, who will implement the relevant aspects of the software within each modality, how often reviews of the collected data and of the program will occur, who is the audience of the reviews, and what the reviews will entail. The review process should also identify the necessary follow‐up actions.

Specific questions include:

1 What is the primary goal of the project, e.g., regulatory or quality improvement (QI)?
2 What will be the reporting structure [Quality Assurance (QA) Committee, Radiation Safety Committee (RSC), other]
3 How can/will the monitored data be used?
4 Which indices are needed for these uses?
5 What do these indices mean?
  - Are they relevant to the overall goal?
  - How accurate are they?
  - How accurate do they need to be?
  - Is one index sufficient?
6 Are the desired indices available for tracking?
7 Where will they be tracked (stored and analyzed)?
8 Are the facility's relevant imaging systems compatible with the RDIM system to send and receive data? If so, how will the data transfer take place?
9 Under what conditions, if any, should either estimated or derived dose quantities or RDI be summed?
10 Who should have access? How much access should they have?

Additional challenges to consider include:

1. Cost and labor involved in set‐up and ongoing support
2. PACS and information technology (IT) issues
3. Testing the software once installed
4. Analyses — population, modality, individual
5. Identifying the type of feedback that will be provided to physicians, (e.g., ordering and protocoling) and the mechanism for communicating that feedback.

### 1.B Goals and rationale

The primary rationale for the acquisition and use of RDIM software is to enhance and expand existing QA and quality control (QC) efforts in a facility. When used for QA purposes, RDIM software may aid a QMP or other user in determining utilization practices by ordering physicians, identifying cases that are outliers compared to other similar cases, studies with parameters outside predefined reference levels, or image‐guided interventions with exposures above a threshold for tissue reactions. With tracking of key patient‐ or study‐specific RDI and imaging parameters, it is possible to enhance existing diagnostic imaging QA programs.^6^ Comprehensive QA programs should address relevant critical aspects of patient imaging including positioning, motion, collimation or imaging extent, and overall image quality. Consequently, QA programs should consider the image acquisition and reconstruction parameters affecting dose and image quality performance across different systems within a given imaging modality, such as the various types of general radiography equipment used within a facility. The ability to capture imaging parameters specific to the individual patient study varies considerably by modality and age of equipment. These will be addressed in the modality‐specific sections below.

Additional approaches to QA are enabled by the use of RDIM software. The automated collection of large numbers of studies from modalities and protocol configurations allows QMPs to identify opportunities for radiation dose optimization, and analysis of practice characteristics by technologist, procedure code, or referring/performing physician, as examples.

### 1.C Potential limitations and precautions

Care should be taken when purchasing RDIM systems to understand the features and capabilities, since some may not be relevant or valid for use in all situations. The appropriate use and interpretation of the data collected and generated by an RDIM system, especially in relationship to patient histories, is an essential role of the QMP. A number of situations describing potential limitations are highlighted below.

#### 1.C.1 Cumulative patient dose history

Assessing risk of deterministic skin injury is the most appropriate use for cumulative dose estimates.^7^, ^8^, ^9^ However, predictions of hypothetical cancer incidence and deaths in patient populations exposed to doses encountered in imaging are highly speculative, may lead to inappropriate medical decision making and thus are strongly discouraged. Cumulative or longitudinal dose values obtained from summing RDI or RDI‐derived quantities for an individual patient indicate stochastic risks that are based on hypothetical cancer incidence or death. Therefore, they should not be used as a basis for decisions regarding subsequent medical radiological procedures.^10^, ^11^

#### 1.C.2 RDIs are not patient dose

It is of utmost importance to understand that the RDI reported by modalities are not accurate reflections of the patient absorbed dose.^1^, ^12^ In certain cases, the use of Monte Carlo dose estimates, size‐based corrections, or peak skin dose estimates may give a reasonable estimate of the patient dose. The methods used to generate these dose estimates must be transparent and based on peer‐reviewed scientific literature. A QMP should be involved in considering the appropriate use and interpretation of the dose estimates.

Furthermore, the summation of modality‐generated RDIs from exposures to different areas of the body, even if the exposures occur during the same examination or within a short amount of time, may not produce a meaningful result.

#### 1.C.3 Patient dose estimates

Consideration and discussion of the stochastic risk associated with the low levels of ionizing radiation to which patients are exposed during diagnostic imaging studies is beyond the scope of this document. The use of effective dose estimates using population‐based tissue‐weighting factors to quantify risk for individual patients is not supported by current science.^2^

There is substantial and convincing scientific evidence for health risks due to tissue reactions^13^ from elevated or high radiation doses. Alert levels can be established by modality, such as interventional fluoroscopy or dynamic computed tomography (CT) acquisitions (perfusion, CT‐guided biopsy, etc.), to flag studies after they are completed for the possibility of tissue reactions. For estimation of tissue reactions, a peak skin dose estimate is usually desired. A QMP should review the individual patient record and the data associated with image acquisition parameters to make such assessments.^7^ RDIM systems may not currently have the ability to provide real time alerts for radiologic examinations that may cause tissue reactions, but can be used to retrospectively monitor such cases and identify patterns or trends. Modern CT scanners and fluoroscopy units include safety features which can identify situations in real time, which may result in tissue reactions, and RDIM systems can be used to complement these modality features.

## 2 Definitions and Acronyms

### 2.A Acronyms and abbreviations

AGD: Average Glandular Dose
CT: Computed Tomography
CTDIvol: Computed Tomography Dose Index – volume
CR: Computed Radiography
DAP: Dose‐Area Product (synonymous with PKA, below)
DI: Deviation Index
DICOM: Digital Imaging and Communications in Medicine
DLP: Dose Length Product
DR: Digital Radiography
DRL: Diagnostic Reference Level
EI: Exposure Index
EMR: Electronic Medical Record
ESAK: Entrance Skin Air Kerma
ESE: Entrance Skin Exposure
FGI: Fluoroscopically Guided Interventional
HIPAA: Health Insurance Portability and Accountability Act of 1996 (HIPAA Pub.L. 104–191, 110 Stat. 1936, enacted August 21, 1996
HIS: Hospital Information System
HITECH: Health Information Technology for Economic and Clinical Health (HITECH) Act of 2009
HL7: Health Level Seven International
IAEA: International Atomic Energy Agency
IEC: International Electrotechnical Commission
IHE: Integrating the Healthcare Enterprise
IT: Information Technology
K_a,r_: air kerma at the reference point^8^
MPPS: Modality Performed Procedure Step
MSK: Musculoskeletal
NM: Nuclear Medicine
OCR: Optical Character Recognition
PACS: Picture Archiving and Communication System
PERP: Patient Entrance Reference Point [cite IEC standard]
PET: Positron Emission Tomography
PHI: Protected Health Information
P_KA_: Kerma‐Area Product (synonymous with DAP, above)
PQI: Practice Quality Improvement
PSD: Peak Skin Dose
QA: Quality Assurance
QC: Quality Control
QMP: Qualified Medical Physicist
RDI: Radiation Dose Index (or Indices)
RDIM: Radiation Dose Index Monitoring
RDSR: DICOM Radiation Dose Structured Report
REM: Radiation Exposure Monitoring
R/F: Radiographic/Fluoroscopic
RIS: Radiology Information System
RRDR: DICOM Radiopharmaceutical Radiation Dose Report
RSC: Radiation Safety Committee
SSDE: Size‐Specific Dose Estimate
SRDL: Substantial Radiation Dose Level
WED: Water Equivalent Diameter

### 2.B Definitions

1. Qualified Medical Physicist (QMP) — as defined by the American Association of Physicists in Medicine (AAPM) Professional Policy 1,^14^ for this practice guideline the applicable subfields are diagnostic and nuclear medicine physics.
2. Radiation Dose Index (RDI) — A RDI is a descriptor of the radiation used to generate images for physician interpretation.

## 3 The Radiation Dose Index Monitoring Software Team

Successful implementation of RDIM software requires a team effort. At a minimum, this team must consist of a QMP, a lead radiologist, a lead technologist, and an individual from the PACS/IT department. The team should include a senior administrator with authority over all departments that use radiation sources for imaging (such as a Chief Medical, Operating, or Administrative Officer, Vice President, or as determined by facility leadership). Alternatively, an administrator (manager, director, etc.) from each such department should participate. In addition, a physician from each other department using radiation‐generating imaging equipment (e.g., cardiology, pain management, neurosurgery, vascular surgery) should participate. At many facilities, the RSO may be a key member of the team.

If the RDIM team does not include a senior administrator, there should be a clear delineation of its reporting structure. Facilities with a RSC should consider having the RDIM team report to the RSC.

This team must be responsible for decisions regarding the selection of the software system. Each team member brings different expertise and may have a variety of responsibilities in the implementation and use of the RDIM software. To be successful, it is very important that the expectations of roles and responsibilities of each member are clearly described. The ability to work together in a team environment will be an important attribute of each member of this group.

## 4 Informatics Recommendations/Requirements

RDI monitoring is fundamentally an imaging informatics and medical physics effort, combining computer science, information technology, and networking with concepts and problems from medical imaging. A viable RDIM system must interface with a number of other clinical imaging information systems, possibly including, but not limited to, PACS, RIS, EMR, voice recognition and dictation systems, critical results reporting systems, and operational and quality dashboards.

Users evaluating RDIM technology should carefully consider a number of informatics‐related system integration factors in selecting a solution and planning their budget and time needs for installation and integration. For example, some commercial RDIM solutions consist of hardware and software, while others consist of software and require the user to furnish server and workstation hardware to host the RDIM solution. The physical and logical architecture of the system must be examined to determine whether there are enough network ports in the right locations to connect all of the nodes that will send data to the RDIM solution or receive information from it. Each imaging modality might allow global configuration (at the imaging system console level) to send the desired type of RDI data to the RDIM system for all examinations, or information transfers may need to be configured within each individual preset imaging protocol. Likewise, each imaging system might automatically transmit the desired RDI data, or specific actions might be required of the operator to send the RDI data/file/object to the RDIM system. On some imaging modalities, the end user may have access to configure network settings and options to send RDI data, while on others, service passwords or special access may be needed to perform such configurations. This is a sampling of issues that require different approaches for the various combinations of RDIM and connected systems, which can lead to significant unforeseen costs and time delays if not planned for appropriately. Facilities should create performance expectations for these issues prior to system selection.

To ensure adequate interconnectivity, data security, and data integrity, the following informatics recommendations and requirements apply to all RDIM systems:

1 RDIM systems must communicate using appropriate DICOM and HL7 standards and conform with appropriate IHE integration profiles (e.g., REM).
2 RDIM systems must provide data portability, meaning that the RDI information database remains the property of and accessible to the healthcare institution (end user) after the termination of any RDIM software subscription or support agreement.
  - All RDI information, patient demographics, and other data transmitted to the RDIM system by the end user's systems must be made available to the end user in a format and medium that may be retained and accessed by the end user without proprietary tools associated with the RDIM system. Data need not be stored in such a format in the RDIM working database; if the RDIM solution uses a proprietary database, export tools or functions must be provided.
  - Data or values that are calculated, simulated, or generated by the RDIM system may be stored or exported in proprietary or nonproprietary formats. Methods by which these values are created must be clearly referenced.
  - User documentation for RDIM systems should include a data portability summary describing the storage format and medium used for each type of data collected (and, if applicable, generated) by the system.
3 Patient PHI transmitted to and stored in RDIM systems is subject to the requirements of the HIPAA and HITECH laws and related regulations. The RDIM user must develop a written plan for data management and security. If PHI will be transmitted to any third party providing or supplying an RDIM solution, a written agreement with this party must be developed to address the data management and security responsibilities of both parties.
  - RDIM system user documentation should disclose the system's data communication and storage topology to the end user to clearly identify all locations (physical, virtual, and logical) used for transmission, receipt, and storage of PHI by the system.
  - Any PHI transferred outside of the user's internal secure network must be encrypted and otherwise secured in accordance with HIPAA and HITECH requirements.
  - Third parties who supply or host RDIM solutions must ensure that any offsite or cloud storage of PHI fulfills the PHI security obligations of the end user's institution providing the PHI, since end users do not have physical control of PHI stored in such locations.
  - Data security for RDIM systems should be implemented and monitored in accordance with the *ACR‐AAPM‐SIIM Practice Parameter for Electronic Medical Information Privacy and* Security.^15^
4 RDIM software capable of receiving, storing, and processing a particular type of RDI data or value should do so with equivalent capability for such data generated by equipment of all manufacturers, models, and software versions for all supported modalities. RDIM software may have capabilities to read or process additional data or proprietary formats from some modalities. RDIM software should not decline, delete, discard, or ignore any data or values provided to it via standards‐compliant interfaces (e.g., DICOM RDSR) or other nonproprietary supported interfaces (e.g., OCR or MPPS). RDIM software may discard (or flag for review) data suspected to be unreliable due to technical limitations of the data input interface (e.g., incomplete or incorrect values from OCR).
5 If review or analysis elements of the user interface, such as plots, charts, or tables, can be exported from the RDIM software, the export should produce a nonproprietary or commonly accessible file type (e.g., BMP, TIF, JPG, PNG, PDF, XML, TXT, CSV, DOC/DOCX, XLS/XLSX).
6 Means for backup of the RDIM data must be provided. If the RDIM software performs backup functions, the RDIM user documentation must describe how to access and restore the backup data. Periodic data backups should be produced automatically. Backup data is subject to the information security requirements and considerations described above for PHI.
7 A variety of means exist for collecting RDI data. Therefore, the end user of the RDIM system should carefully assess the type(s) of data that can be sent by each imaging device to be monitored and ensure that the RDIM system supports one or more data collection methods for all imaging devices. Examples of methods for RDI data collection are:
  - DICOM Radiation Dose Structured Report (RDSR) objects
  - DICOM Modality Performed Procedure Step (MPPS) messages
  - As described by IHE Radiation Exposure Monitoring (REM) profile
  - Screen capture images of “dose reports” or “protocol information” with extraction via Optical Character Recognition (OCR)
  - Manual entry
  - Protocol and/or dose information stored in DICOM image file headers
8 Prospective users of RDIM technology should carefully consider the following in selecting a solution and planning budgets, installation, and integration:
  - Determine whether the required workstation or server hardware is user‐supplied or sold in a package with the software.
  - Given the physical location for installing the servers and routers, ensure that there is appropriate network connectivity, including wireless devices, for all locations to be connected. This includes imaging equipment as well as HIS, EMR, RIS, PACS, dictation/reporting software, and any other systems or devices that may need to be connected to the RDIM system.
  - Determine whether each individual imaging system allows global configuration to send the desired data to the RDIM system for all examinations, or whether the information transfers need to be configured within each individual imaging protocol.
  - Determine whether each imaging system automatically transmits the desired data or whether specific actions must be taken by the operator to send the RDI data/file/object to the RDIM system.
  - Determine whether the end user has the ability to configure the required connectivity settings or options on each imaging modality, RIS, PACS, or other connected system. Service engineers, applications specialists, or IT support personnel may need to be engaged to configure some transfers.

## 5 Recommended Elements Common to All Dose Tracking Software Independent of Modality

The following elements apply to all RDIM software and are independent of the specific imaging modality. As an essential tool for improving a QA program and ensuring regulatory compliance, RDIM software should provide users several fundamental functions that are available for all imaging modalities. (Table 1)

**Table 1:** Elements common to all modalities

Elements	Description
Essential RDI	Essential RDI of the imaging modality must be recorded. If not automated, essential acquisition parameters must be able to be recorded with manual input capability.
Notifications for RDI outside a defined range	User‐defined thresholds of RDI that trigger automatic notifications to a set of end users must be configurable in the RDIM software.
Review/follow‐up documentation	User inputs for status tag and notes must be provided so that the user who reviews the alert can document acknowledgement of each alert and the status and outcome of any follow‐up in the RDIM software.
User management	Access to the RDIM software must be limited to a group of authorized users as determined by the facility.
Audit trails	User identity, date and time stamp, and details of activity must be logged for all manual data inputs, edits, and deletions performed by RDIM software users.
RDI analysis tools	RDI analysis tools should be provided to assist users in utilizing the collected information.
User interface elements	A user interface should provide key functionalities of reviewing the recorded RDI and imaging acquisition parameters.

### 5.A Common elements that are independent of modality

#### 5.A.1 Tracking of RDI

For each imaging modality, its corresponding essential RDI must be recorded. There are often multiple irradiation events in a single patient examination (e.g., multiphase CT scans, irradiation from multiple views in fluoroscopic and other X‐ray exams). The RDI of each irradiation event, including live fluoroscopy and rejected images must be recorded unambiguously if that information is transmitted to the RDIM software. In addition, available essential acquisition parameters should be recorded together with the corresponding RDI data. If the RDI information and/or acquisition parameters are not automatically recorded, the option of manual input of such information by the user must be provided.

#### 5.A.2 Notifications for RDI outside the defined range

It is often necessary to preset thresholds of RDI for different purposes, such as quality/safety assurance and regulatory compliance. The RDIM software must allow users to set thresholds of RDI which may be based on a variety of criteria, including but not limited to modality, type of examination, and patient age. The users can determine the thresholds of RDI based on either their institution specific experience or publicly available resources such as published DRLs. In the scenario where the RDI of an examination exceeds its threshold, the predetermined target users must be notified in a timely manner. If protected health information (PHI) is included in the notification, it must be transmitted using a secure, HIPAA‐compliant approach.

#### 5.A.3 Review or follow‐up documentation

For each alert generated, the RDIM software must provide a means for users to document that the alert has been acknowledged, reviewed, or followed‐up as appropriate. This should include a status flag or tag for each alert, so that alerts can be summarized by categories such as Needs Review, Under Review, Pending Follow‐Up, Closed, etc. Each alert should also have input and storage for free‐text notes to store information related to the alert and the follow‐up. Since notes may contain PHI, review and follow‐up documentation must be stored in a secure, HIPAA‐compliant manner.

#### 5.A.4 User management

Access to the RDIM software must be limited to a group of authorized users, whose access is password protected. The level of data access and system configuration must be granted according to the specific role of each user or group of users as determined by the RDIM team.

#### 5.A.5 Audit trails

For any manual action by a user that modifies the RDIM data (e.g., manual input, editing, deletion, adding notes, setting or clearing alerts or flags), the RDIM software must log the identity of the logged‐in user account, date and time stamp, and information about the nature of the manual data activity in an audit trail. For operations that edit or delete data, the RDIM software should retain a copy of the original data so that the change can be reverted if necessary.

#### 5.A.6 RDI analysis tools

The software should provide users tools to utilize the RDI information to assist with continuous practice quality improvement. Meaningful ways to analyze dose information should include but not be limited to comparing RDI of user‐selected protocols across machines, analyzing the trending of RDI as a QA tool and reviewing patient history which could include examinations from multiple different imaging modalities.

#### 5.A.7 User interface elements

A user interface should provide a variety of functionalities for users to review the recorded RDI data, including but not limited to navigating examinations with customizable sorting options (e.g., chronologically, alphabetically, or by age), exploring detailed examination and RDI information of any user‐selected examination, reviewing examination history of any user‐selected patient, categorizing RDI data by modality, facility, individual device, date range, examination type, protocol type, ordering physician, performing physician, or operating technologist. The function of exporting RDI data should be provided and the format of exported data should be compatible with commonly used analysis software (e.g., Excel). A note function that allows a user to attach text notes to various locations in the database and/or user interface should be included. Such notes are useful for capturing information about reviews of data other than alerts. For example, users may find it beneficial to attach notes to a patient, examination, individual exposure event, or to a chart, plot, or table in the data review/analysis user interface.

The essential RDI for monitoring and notification for each modality are discussed in the modality‐specific sections below.

## 6 Recommended Elements Specific to Computed Tomography (CT)

CT provides the largest overall contribution to population radiation dose from medical imaging.^16^ CT has been a particular area of focus for RDIM software^17^, ^18^ in part due to the number of highly publicized cases in which excessive amounts of radiation were delivered in CT examinations.^19^, ^20^ It is especially important to remember that the RDI associated with CT examinations are not the patient dose.

The minimum required elements apply to the collection of RDI from 3rd generation or “rotate‐rotate” CT systems such as those found in diagnostic radiology departments, integrated with SPECT or PET systems and CT simulation systems found in Radiation Oncology departments. The following table of software features describes the minimum recommended elements for RDIM of diagnostic CT examinations. The features are explored in greater depth below. (Table 2)

**Table 2:** Elements specific to CT

Elements	Description
Essential RDI	CTDIvol, CTDI phantom, and DLP for each CT series must be recorded.
Notification of RDI outside of defined range	User‐defined thresholds of RDI that trigger automatic notifications to a set of end users must be configurable.
Transmission of anonymized data to data repositories/registries	The software must possess the capability to transmit CT RDI to data repositories/registries.
Size‐Specific Dose Estimate (SSDE) calculation	Calculation of the SSDE for applicable acquisitions should be available.

### 6.A Elements specific to CT

#### 6.A.1 Automatic monitoring of essential dose metrics

The RDIM software must be able to record the CTDIvol, DLP, and the size of the CTDI phantom used to calculate these values (if provided) from the appropriate DICOM fields for each ionizing radiation event. If other RDI are available, the software should be able to automatically extract that information as well and associate information with the examination in the database.

#### 6.A.2 Notification of dose metrics outside of a defined range

The RDIM software must allow the user to define thresholds for the monitored RDI. Additionally, the software must have the capability to alert a set of users when a RDI from a completed study exceeds the threshold. The software must allow thresholds to be defined at a minimum for CTDIvol and DLP, and should allow definition at a more granular level, such as by protocol, series, body part imaged, or patient age.

#### 6.A.3 Transmission of anonymized data to repositories/registries

The RDIM software must be able to submit the relevant RDI and study information to an external data repository or registry. The software must be able to either transmit anonymized examination and RDI data directly to the repository/registry or communicate the examination and RDI data to an intermediary software program used for anonymization and transmission.

#### 6.A.4 SSDE calculation

AAPM Report 204 defines the Size Specific Dose Estimate (SSDE) as a RDI in CT that takes into account patient size and the reported CTDIvol to provide an estimate of the patient absorbed dose for a CT acquisition.^21^ AAPM Report 204 defines conversion coefficients between CTDIvol and SSDE for patient effective diameters, and AAPM Report 220 defines a number of different approaches to calculate that patient water equivalent diameter (WED) which is equivalent to the effective diameter used in AAPM Report 204.^22^ RDIM software should be able to use the information provided in the AAPM reports to calculate the SSDE for specific CT acquisitions. This function may be limited or unavailable if the full field of view reconstructions are not available (such as CTs of the spine or MSK studies). If SSDE is reported by the RDIM software, the calculated WED and the methodology employed to calculate the WED should also be available to the user.

If the modality includes a SSDE value in the DICOM metadata of a study, the software should be able to extract that information and WED and associate it with the examination in the database.

## 7 Recommended Elements Specific to Fluoroscopy

The minimum requirements listed for fluoroscopy are applicable to all fluoroscopy‐guided interventional (FGI) suites, general R/F rooms and mobile fluoroscopy units. Fluoroscopy time alone is not an adequate quantity for assessing deterministic or stochastic risk to the patient.^23^ General convention is to utilize air kerma at the reference point (K_a,r_)^8^ to assess examinations for deterministic risks such as skin erythema and desquamation, epilation, or dermal atrophy, and to use kerma‐area product (P_KA_) for stochastic risk assessment.^24^, ^25^ For this reason, examination time, K_a,r_ and P_KA_ must be recorded, and when possible detailed information for each irradiation event of a procedure (e.g., fluoroscopy as well as 2D and 3D acquisition events) should be recorded so a more thorough assessment of peak skin dose (PSD) can be performed. Given that the displayed K_a,r_ may be inaccurate by as much as ±35%, a QMP should assess whether a correction factor should be applied to the displayed K_a,r_.^26^, ^27^ RDIM software should allow for unit‐specific (by imaging system) correction factors to be assigned for accurate reporting of cumulative K_a,r_. (Table 3)

**Table 3:** Elements specific to fluoroscopy

Elements	Description
Essential RDI	Fluoroscopy time, K_a,r_ and P_KA_ must be recorded; for bi‐plane imaging systems, RDI must be recorded separately for each X‐ray tube (e.g., A/B or PA/lateral). Number of irradiation events and acquisition details (i.e., kV, filtration, mA, number of frames, gantry angle, etc.) should be recorded.
Manual entry of RDI data and fluoroscopy time	Manual entry of fluoroscopy time and RDI data should be available for systems with no RDI information displays or those which have no ability to transfer such data electronically.
Exposure incidence map	A graphical indicator of cumulative K_a,r_ distribution across a two‐dimensional plane that intersects the patient entrance reference point (PERP), potentially highlighting areas of peak air kerma, which can be used to estimate peak skin dose (PSD)^7^ should be available for systems connected using RDSR.
Notification of RDI outside of defined range	User‐defined thresholds of RDI that trigger automatic notifications to a set of end users must be configurable.

### 7.A Elements specific to fluoroscopy

#### 7.A.1 Automated monitoring of essential RDI

The software should be able to extract fluoroscopy time, K_a,r_, P_KA_, and total number of irradiation events from the appropriate structured fields. When available, the software should also automatically extract technical factors for each event acquisition log (i.e., RDI and system geometry for fluoroscopy as well as 2D and 3D acquisition event). Acquisition (or exposure or run or serial fluoroscopy) logs provide a line‐by‐line review of the stored exposures. Fluoroscopy system vendors vary in the extent of information provided, including but not limited to the individual event(s) exposure time, tube position, tube angle, mode, filtration, and K_a,r_. Acquisition logs can be utilized by a QMP to assess cumulative and peak skin doses.^7^

#### 7.A.2 Manual entry of RDI

For older fluoroscopy units, RDI may not be available or the system may lack the capability to export data to the RDIM server. In these instances, at minimum, a record of fluoroscopy time may be sufficient to meet federal and state requirements, and would require manual entry by the user. This option must be available.

#### 7.A.3 Exposure incidence map

The DICOM RDSR or acquisition logs (with line‐by‐line history of the tube position and individual event cumulative K_a,r_) should be utilized to create an incidence map that can more appropriately identify the peak air kerma value. The peak air kerma value as reported by the vendor can then be used by a QMP to assess the risk of tissue reactions of the procedure by estimating the PSD.^7^, ^22^

#### 7.A.4. Notification of RDI outside of defined range

NCRP Report 168^8^ outlines recommendations for patient radiation dose‐management programs and setting substantial radiation dose levels (SRDL) that initiate additional dose‐management procedures (e.g., patient follow‐up visits with the provider or primary care physician). In addition to SRDLs, regulatory and accrediting bodies outline requirements and recommendations for single incidence and longitudinal tracking of patient cumulative skin dose over specified periods of time (e.g., sentinel events). RDIM software must identify single events and cumulative exposures that exceed K_a,r_ thresholds for regulatory and site‐specified SRDLs.^3^

## 8 Recommended Elements Specific to Projection X‐ray (includes mammography and CR/DR)

This section applies to all projection radiographic X‐ray imaging systems including general radiographic units with computed radiography (CR) or DR image receptors, cephalometric units, as well as mammographic systems.

Entrance skin air kerma (ESAK, or entrance skin exposure, ESE) has been a prevalent RDI for many years. Many regulatory agencies continue to use ESE or ESAK as a benchmark and set either recommended or mandatory limits based on this quantity. Historically, the medical physicist has calculated annually the ESE or ESAK for a facility's typical radiographic views on each system.

The introduction of transmission ionization chambers and mathematical algorithms has made available patient‐specific ESAK values as well as P_KA_. Digital mammography systems compute and provide phantom Average Glandular Dose (AGD) values and often ESAK values as well.

The replacement of film with CR and DR technology has created the need for image receptor exposure metrics such as the IEC Exposure Index (EI) and the related Deviation Index (DI).^28^ Image receptor vendors originally developed proprietary exposure indices to meet this requirement. Newer equipment is being designed to comply with the IEC standard Exposure Index definition.^28^

Monitoring of RDI for projection X‐ray imaging is important for identifying outlying exposure events and, where applicable, identifying exposure events that fail to comply with regulations or accreditation requirements. (Table 4)

**Table 4:** Elements specific to projection radiographic X‐ray AGD, Average Glandular Dose

Elements	Description
Essential RDI	KAP, K_a,r_, and AGD (mammography only) must be recorded if available.
Exposure indices	Exposure indices, whether the standard IEC definition or manufacturer‐defined, must be imported and recorded.
Notification of RDI outside of defined range	User‐defined thresholds of RDI that trigger automatic notifications to a set of end users must be configurable.

### 8.A Elements specific to projection radiographic X‐ray

#### 8.A.1 Automatic monitoring of essential RDI

The software must be able to extract KAP and reference air kerma (if available) for general radiography exposures. Capture of machine technical factors and patient demographics is also desirable. For mammography, at a minimum the software should be capable of extracting phantom AGD and compressed breast thickness.

#### 8.A.2 Exposure indices

##### IEC standard

In addition to RDI, the RDIM system must be able to capture image receptor exposure indicators. For CR and DR systems that have adopted the IEC standard,^28^ the software must allow entry of protocol‐specific target indices and protocol‐specific acceptable values for DI, and must allow alert levels to be applied to imported DIs falling outside the acceptable values, as described below in iii.

##### Manufacturer‐defined

For systems that employ proprietary EIs, the software should allow capture of these indices as well as the ability to apply alert levels as described below in iii. Systems that utilize nonstandard exposure indices might not employ a DI. In these cases, the alert values must allow for specification of both a high and low limit.

#### 8.A.3 Alert levels

All stored RDI should have associated with them the ability for the user to assign alert values. Alert values are often single threshold values for RDI, but for projection image exposure indices (EI, DI, etc.) the alerts should be based on acceptable ranges established by the facility's QA program. Alert ranges must be established by QMP, and must be unique to the examination, view, and patient habitus.

## 9 Nuclear Medicine (includes PET)

The collective dose from nuclear medicine (NM) procedures increased approximately sevenfold between 1980 and 2006 with an estimated per capita effective dose of 0.80 mSv, second only to that from CT.^15^, ^29^ This increase is due primarily to the emergence of myocardial perfusion imaging for coronary artery disease screening, which is now ubiquitous. Additionally, ^18^F‐FDG PET is now a mainstream imaging modality in the oncology setting (diagnosis, staging/re‐staging, patient treatment management). Bone scanning with either ^99m^Tc‐labeled agents or^18^F sodium fluoride (NaF), continues to be a commonly employed procedure, again primarily in the oncology setting. NM is also seeing increased use in the pediatric population, driven primarily by the use of ^18^F‐FDG in pediatric oncology.^29^ As a consequence, monitoring of radiation exposure from NM procedures as well as dose reduction strategies have become important concerns in the NM community (Table 5).^30^, ^31^, ^32^

**Table 5:** Elements specific to nuclear medicine

Elements	Description
Essential RDI and RDI‐related parameters	The software must record all essential RDI‐related parameters. See 9.a below for list of elements specific to nuclear medicine.
Manual entry of RDI and RDI‐related parameters	The software must support manual entry of procedure and patient RDI‐related parameters.
Multiple radiopharmaceutical administrations	The software must support single procedures that involve multiple radiopharmaceutical administrations.
Organ and effective dose estimates	The software should support calculation of reference organ and effective dose estimates.
Notification of RDI outside of defined range	User‐defined thresholds of RDI that trigger automatic notifications to a set of end users must be configurable.

### 9.A Elements specific to nuclear medicine

#### 9.A.1 Automated monitoring of essential RDI and RDI‐related parameters

The patient demographic data listed below may be automatically included in all imaging procedures stored in the RDIM software, depending on how the various hospital informatics and modality systems are configured to communicate with each other and the RDIM software. Specifically, for NM the patient demographics must be included in the exam data, because the estimated dose for a given radiopharmaceutical depends not only on the administered activity, but also on the patient's gender, age (e.g., pediatric versus adult) and body habitus. The software must support automatic recording of the following essential NM RDI‐related parameters, and should support DICOM RRDR:

1. Procedure Name
2. Radionuclide
3. Radiopharmaceutical
4. Preadministration Activity
5. Postadministration Residual Activity
6. Dates/Times of Activity Assays
7. Activity Administration Date/Time
8. Route of Administration
9. Patient gender, age, height, and weight at time of exam

#### 9.A.2 Manual entry of RDI

The software must support manual entry of essential NM RDI and the RDI‐related parameters listed above.

#### 9.A.3 Multiple radiopharmaceutical administrations

There exist procedures that involve two radiopharmaceutical administrations (e.g., stress‐rest myocardial perfusion, simultaneous solid‐liquid gastric emptying). The software must be able to account for these multiple radiopharmaceutical procedures.

#### 9.A.4 Organ and effective dose estimates

It is standard practice to provide estimates of both organ and effective doses associated with NM procedures. The software should support calculation of organ and effective dose indices for a reference phantom or population representative of the patient, taking into account the patient's gender and age at the time of the procedure. Such estimates are only appropriate for diagnostic procedures. The estimates should be based on appropriate and recognized published reference tables. The software may support patient‐specific dose calculations using established and/or peer‐reviewed methods (e.g., Monte Carlo or deterministic transport, convolution). The reference(s) upon which the dose estimates are based should be clearly cited on each report. For some radiopharmaceuticals, multiple reference tables exist that account for different primary organ uptake‐dependent biodistribution, such as those for iodine‐radiolabeled sodium iodide (NaI), where separate tables exist for different nominal percent thyroid uptakes. There may also be diagnostic procedures where a reference is not available for dose estimates. In such cases, the software may support user entry of dose tables. Entry of dose tables must be performed by a QMP and should indicate the citable source for the data.

#### 9.A.5 Notification of RDI outside a defined range

No globally accepted DRLs for NM procedures based on either published RDI ranges or regulatory limits exist at the time of publication of this practice guideline. However, guidelines for DRLs for a number of NM procedures have been published by advisory bodies and professional organizations (e.g., NCRP, American College of Radiology, Society of Nuclear Medicine and Molecular Imaging, and American Society of Nuclear Cardiology).^33^, ^34^ The RDIM software must allow the user to define thresholds for the monitored RDI for each procedure. Additionally, the software must have the capability to alert a set of users when a RDI from a completed procedure exceeds a defined threshold. The software must allow thresholds to be defined for either activity or dose, and should allow definition at a more granular level, such as by organ, or patient gender or age.

## 10 Conclusions

Radiation dose index monitoring (RDIM) systems are being developed and implemented in response to concerns about the increased utilization of and patient population exposure to ionizing radiation from medical diagnostic imaging devices and associated regulatory dose reporting requirements. This report discusses important issues and features to consider when evaluating RDIM systems for purchase and implementation. These systems have potential to revolutionize QA in diagnostic imaging and present unique research opportunities. The data from RDIM software may be useful in assisting the QMP in such tasks as ongoing QA, PQI projects, and patient or fetal dose estimation. However, the data must be aggregated and derived in a transparent manner by the manufacturers and handled responsibly by the imaging facility and QMP.

## Conflict of interests

The authors declare no conflict of interest.
